# Radical-assisted chemical doping for chemically derived graphene

**DOI:** 10.1186/1556-276X-8-534

**Published:** 2013-12-19

**Authors:** Ryousuke Ishikawa, Pil Ju Ko, Masashi Bando, Yasuyoshi Kurokawa, Adarsh Sandhu, Makoto Konagai

**Affiliations:** 1 Department of Electrical and Electronic Engineering, Tokyo Institute of Technology, 2-12-1 O-okayama, Meguro, Tokyo 152-8552, Japan; 2 Electronics-Inspired Interdisciplinary Research Institute (EIIRIS), Toyohashi University of Technology, 1-1 Hibarigaoka, Tempaku-cho, Toyohashi, Aichi 441-8580, Japan; 3 Department of Physical Electronics, Tokyo Institute of Technology, 2-12-1 O-okayama, Meguro, Tokyo 152-8552, Japan; 4 Photovoltaics Research Center (PVREC), Tokyo Institute of Technology, 2-12-1 O-okayama, Meguro, Tokyo 152-8552, Japan

**Keywords:** Chemically derived graphene, Transparent conductive films, Carrier doping, Charge transfer, Radicalized organic conductors, First-principles calculation

## Abstract

Carrier doping of graphene is one of the most challenging issues that needs to be solved to enable its use in various applications. We developed a carrier doping method using radical-assisted conjugated organic molecules in the liquid phase and demonstrated all-wet fabrication process of doped graphene films without any vacuum process. Charge transfer interaction between graphene and dopant molecules was directly investigated by spectroscopic studies. The resistivity of the doped graphene films was drastically decreased by two orders of magnitude. The resistivity was improved by not only carrier doping but the improvement in adhesion of doped graphene flakes. First-principles calculation supported the model of our doping mechanism.

## Background

Graphene, a single atomic layer of sp^2^ graphitic carbon, has received a lot of attention because of its attractive electromechanical properties and its potential applications for the ‘next-generation’ electronic devices [1-5]. Although mechanically cleaved graphene exhibits excellent electrical performance, such as a highest carrier mobility of over 200,000 cm^2^ · V^-1^ · s^-1^[6]. The rate of production when using this mechanical exfoliation method is extremely limited. Therefore, there has been considerable impetus to discover a scalable production technique. Among the possible candidates, a chemical exfoliation method based on a liquid process is considered to now be well established. One of the greatest advantages of the chemical exfoliation method is that chemically derived graphene can be deposited or formed into films on any large-area substrate [7,8]. Ease of modification and/or functionalization of the graphene are also reasons why the chemical method is widely accepted [9,10]. Furthermore, it has been focused on as a new tunable platform for optical and other applications [11-14].

Carrier doping is a common approach to tailoring the electronic properties of semiconductor materials. Carrier doping can also dramatically alter the electrical properties of graphene. Although several techniques aimed at the carrier doping of graphene have been demonstrated, including boron- or nitrogen-substitutional doping [15,16], the deposition of alkali metal atoms [17], and the adsorption of gaseous NO_2_[18], these doping methods have never achieved significant doping effects due to defect formation, inhomogeneous deposition, and the instability of gaseous species, respectively. Molecular doping, such as halide [19,20] or polymer [21,22], is a promising technique for pristine graphene films. However, effective doping method for chemically derived graphene has never been demonstrated. Tetracyanoquinodimethane (TCNQ) is well known as a powerful electron acceptor, and is expected to favor electron transfer from graphene into TCNQ molecules, thereby leading to p-type doping of graphene [23,24]. Conjugated organic molecules such as these have been widely used in organic light-emitting diodes to improve device performance by controlling the hole injection barrier [25]. Efficient doping of organic semiconductors, of carbon nanotubes, and of graphene has been demonstrated.

We demonstrate herein a novel carrier doping method for chemically derived graphene using radical-assisted conjugated organic molecules in the liquid phase. It is expected that liquid-phase chemical interactions between graphene and conjugated organic molecules induce high doping efficiency. Absorbance measurements provide direct evidence for charge-transfer (CT) interactions between graphene and radicalized TCNQ molecules in an organic solvent. Raman spectroscopy and ultraviolet photoelectron spectroscopy (UPS) have also been used to elucidate the effects of doping on doped graphene films, which showed improvements in resistivity of two orders of magnitude with highly stable doping effect. Previous attempts at carrier doping for chemically derived graphene have never decreased the resistivity by more than one order of magnitude [26]. The doping mechanism of the chemical doping is investigated using first-principles calculation based on density functional theory. Our doping method is compatible with the wet production technique of chemical-exfoliated graphene. The doped graphene films can be formed by the all-wet process via the radical-assisted chemical doping method as demonstrated in this work.

## Methods

### Preparation and reduction of graphene oxide

Chemically derived graphene was synthesized using a modified version of Hummer's method, a well-known approach to producing monolayered graphene via the liquid-phase exfoliation of graphite oxide, as described previously in the literature [27]. Natural graphite powder was donated by SEC Carbon Ltd. (Tokyo, Japan). All other chemicals were purchased from Kanto chemical Co. Ltd. (Sakado, Japan) and used directly without further purification. Chemically derived graphene was synthesized by the modified Hummer's method, a well-known approach to produce monolayered graphene via liquid-phase exfoliation of graphite oxide. Natural graphite powder (SEC Carbon SNO-30) was oxidized in KMnO_4_ and H_2_SO_4_. After centrifugation, the resulting graphite oxide was exfoliated into graphene oxide (GO) by ultra-sonication (100 W, 30 min, 60°C). Then, a GO aqueous dispersion was produced by centrifugation and dialysis to neutralize a pH.

A reduction step of GO into graphene plays an essential role to determine the electrical properties of the resulting graphene films. GO was reduced as follows: GO was dispersed in aqueous solution containing N_2_H_4_, a strong reductant, with NH_3_ to adjust pH. The mixed solution was reacted in a water bath at 95°C for 1 h, and the color of dispersion changed from brownish color to gray. Finally, the solvent of reduced graphene oxide (RGO) dispersion was replaced by *N*,*N*-dimethylformamide (DMF) using an evaporator. RGO can be dispersed well in many kinds of organic solvents including DMF, while it is easily aggregated in aqueous solution because of its low electrostatic repulsion force.

### Doping and film fabrication

Doping graphene via charge transfer by TCNQ molecules was carried out as follows. First, 0.01 g of TCNQ powder (>98.0%, Tokyo Chemical Industry Co. Ltd., Tokyo, Japan) was dissolved into 5 ml of DMF solvent. Then, 5 ml of RGO dispersion and radicalized TCNQ in DMF were mixed and stirred for 1 week at room temperature. The color of mixture solution changed from yellow-green to orange. Our graphene films were deposited on glass substrates (Corning7059) by a spray coat method at a substrate temperature of 200°C in an atmosphere containing the solvent vapor. The thickness of the films was controlled by varying the spray amounts.

### Characterization

The Raman spectroscopy was measured with a Jasco NRS-1000 (excited by a 532-nm green laser; Easton, MD, USA). Absorbance and transmittance spectra were obtained with Shimadzu SolidSpec3700 UV–vis by using a quartz cell for absorbance measurements. The sheet resistance was measured by van der Pauw method at room temperature in air. The presence of monolayered GO flakes in our synthesized GO aqueous solution was verified by atomic force microscope images by Raman peak shifts and by the peak shape of the second-order two-phonons process peak at 2,700 cm^-1^, referred to as the 2D band. The size of the flakes is up to 50 × 50 μm^2^. After liquid phase reduction by N_2_H_4_ and NH_3_, the solvent of the RGO aqueous solution was replaced by DMF using an evaporator. RGO can be dispersed well in many kinds of organic solvents including DMF, while it is easy to aggregate in aqueous solution due to its low electrostatic repulsion force. The conductivity and the Hall carrier mobility of individual monolayered RGO flakes were as high as 308 S · cm^-1^ and 121 cm^2^ · V^-1^ · s^-1^, respectively. Hall measurements were conducted in air at room temperature using Hall-cross geometry and Au/Ti electrodes.

### Calculation details

The electronic structural analysis is carried out using the SIESTA3.1 code, which performs fully self-consistent calculations solving the Kohn-Sham equations [28]. The Kohn-Sham orbitals are expanded using linear combinations of pseudo-atomics orbitals. The double-zeta polarized (DZP) basis set was chosen in this study. The calculations were done with the local density approximation (LDA), using the Ceperley-Alder correlation as parameterized by Perdew and Zunger [29]. The electron-ion interaction was treated by using norm-conserving, fully separable pseudo-potentials [30]. A cutoff of 200 Ry for the grid integration was utilized to represent the charge density. Two TCNQ molecules on and under the (4 × 4), (6 × 6), or (8 × 8) graphene supercell units were simulated for full relaxation of the systems. The Brilliouin zone was sampled by 20 × 20 × 1 ***k***-points using the Monkhorst-Pack scheme for electronic properties calculations. It is necessary to ensure that the *z* axis of the periodic supercell (normal to the graphene surface) is large enough so that there is negligible interaction between the two graphene sheets. A distance of 170 Å along the *z* axis is found to be sufficient to ensure the energy convergence for configurations.

## Results and discussion

**Figure 1:**
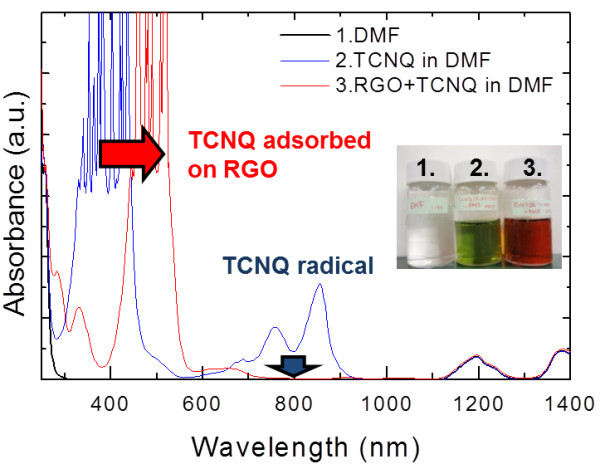
**Absorbance spectra of RGO + TCNQ mixture solution (red line) and radicalized TCNQ solution (blue line).** The inset image shows a photograph of DMF (colorless), TCNQ in DMF (yellow-green), and a RGO + TCNQ mixture solution (orange), respectively. The absorption peak at around 800 nm in the spectrum of TCNQ, which is derived from the TCNQ radical species in the DMF network, had disappeared in the spectrum of the RGO + TCNQ mixture. Additionally, the strongest absorption peak at 400 nm shifted up to 500 nm after the reaction with RGO.

Doping of graphene via CT by using TCNQ molecules was carried out as follows: first, TCNQ powder was dissolved into DMF solvent. It is expected that TCNQ molecules in DMF will be radicalized [31]. Then, the RGO dispersion (0.25 wt.%) and the radicalized TCNQ in DMF were mixed and stirred for 1 week at room temperature. This RGO-TCNQ mixture dispersion was very stable over a few months, and there was no clear evidence of aggregation. We observed the absorbance spectra of this mixture dispersion to investigate CT interactions between RGO and TCNQ in a solvent (Figure 1). The absorption peak at about 800 nm in the spectrum of TCNQ (shown in blue), which comes from the TCNQ radical species in the DMF network, disappeared in the spectrum of the RGO + TCNQ mixture (shown in red). In addition, the strongest absorption peak at 400 nm shifted to 500 nm after the reaction. Such a red shift is also observed in TCNQ with coal aromatics systems [31]. This peak shift was supported by a color change of mixture solution from yellow-green to orange, as shown in the picture inset in Figure 1. These spectral changes indicate that radicalized TCNQ molecules in the DMF network were almost all adsorbed on the RGO flakes and induced the CT interaction.

**Figure 2:**
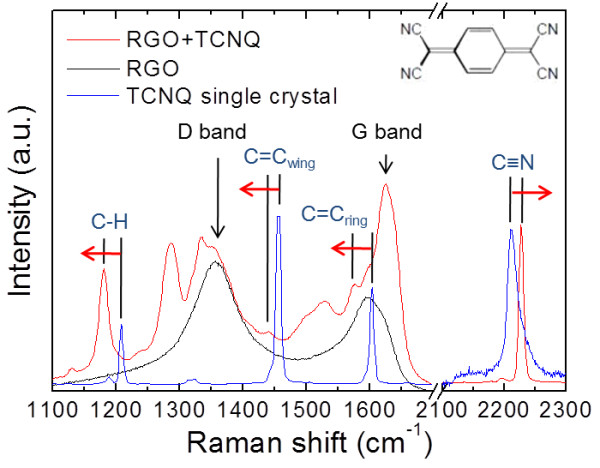
**Raman spectra of fabricated films.** From RGO + TCNQ complex film (red line), RGO film (black line) and TCNQ single crystal (blue line) with an image of TCNQ molecular structure. The Raman spectrum of the RGO + TCNQ complex consists of peaks from TCNQ and RGO (and other unknown peaks). The shifts in the Raman peaks from the TCNQ in RGO + TCNQ complex indicates a charge transfer interaction.

**Figure 3:**
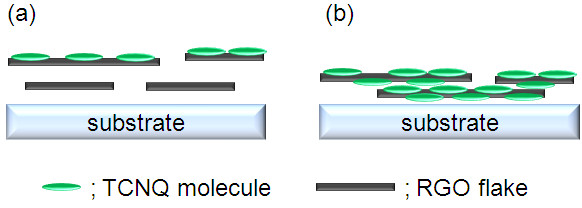
Schematic images of doped RGO films by surface adsorption (a) and RGO + TCNQ complex films (b).

We made an attempt to conduct a Raman spectroscopic study of RGO + TCNQ films fabricated by spray coating and of TCNQ single crystals in order to elaborate the CT interaction. The obtained Raman spectra are summarized in Figure 2. The Raman spectrum of the TCNQ single crystal exhibited the stretching vibration modes of C ≡ N (2,227 cm^-1^), C = C_ring_ (1,603 cm^-1^), and C = C_wing_ (1,455 cm^-1^), and a bending vibration mode of C-H (1,207 cm^-1^). We observed all of the Raman peaks originating from TCNQ molecules in the spectrum of the RGO + TCNQ complex. However, these peaks shifted from those of the TCNQ single crystal relative to each other. The C = C_ring_ stretching and C-H bending vibrations that appeared at 1,580 cm^-1^ and at 1,179 cm^-1^ respectively were similar to the vibration modes in a benzene ring. This indicated that the quinoid ring of the TCNQ molecules transformed to a benzene ring after CT, as in the case of adsorbed TCNQ on single-wall carbon nanohorns [32]. Meanwhile, the C ≡ N stretching vibration shifted up to 2,210 cm^-1^ in the RGO + TCNQ complex sample. The degree of charge transfer, *Z*, was estimated at 0.39 from the C ≡ N vibration frequency, which should be a linear function of *Z*[33]. Moreover, we also examined doping effect from surface adsorption by immersing pristine RGO films in a TCNQ dispersion for comparison [34]. The sheet resistance was also improved because the surface electrons of the RGO film were withdrawn by adsorbed TCNQ molecules, as represented in Figure 3a. The *Z* value (degree of CT) was estimated at 0.27 from the C ≡ N vibration frequency in the Raman spectra. Doping effects from the surface adsorption were limited by the amount of adsorbed molecules, due to the strong intermolecular repulsive interaction [35,36]. On the other hand, our RGO + TCNQ complex films, which are shown as a schematic image in Figure 3b, were improved in terms of sheet resistance from those in previous reports [19,21,26]. It is expected that the notable doping effect was principally achieved by the strong mutual reaction between radicalized TCNQ molecules and RGO flakes in the liquid phase, as predicted from the absorbance spectra. Furthermore, the TCNQ-RGO interaction might accelerate and improve the stacking of films during film fabrication [35,37]. We presumed that these phenomena supported the existence of a high doping effect and a high degree of charge transfer (*Z* = 0.39).

**Figure 4:**
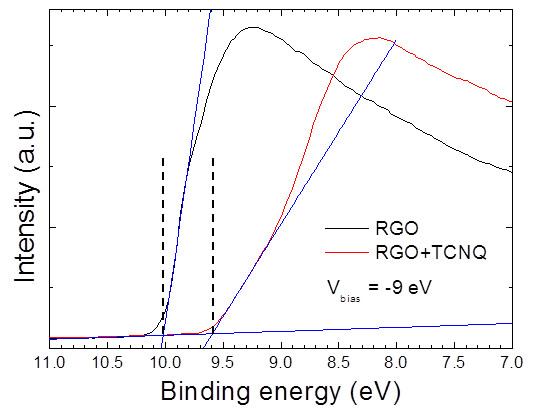
Secondary electron cut-off region UPS spectra of doped and non-doped RGO films.

Additional evidence for the CT interaction was obtained via UPS using He1 radiation (*hν* = 21.2 eV). We measured the UPS spectra of doped and non-doped RGO films under an applied sample bias voltage of -9 eV. The work function (Φ) increased by 0.4 eV from pristine RGO films relative to the RGO + TCNQ films as shown in Figure 4. The change in the surface work function (ΔΦ) might be mainly caused by the Fermi level (*E*_*F*_) shifting towards the Dirac point (*E*_*D*_) due to hole doping from TCNQ via CT, and the interface dipole effect for the TCNQ + RGO films might be smaller than that induced at a deposited F4-TCNQ/graphene interface [34,38].

**Figure 5:**
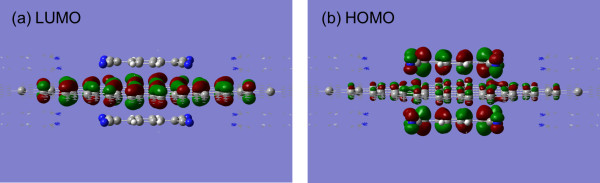
**Plots of wave functions of LUMO and HOMO levels. (a)** Plot of the wave function of the LUMO level in TCNQ/graphene system at Γ point. LUMO is delocalized over π orbitals of graphene. **(b)** Plot of the wave function of the HOMO level shows strong localization on TCNQ molecule. Red and green lobes are of equal amplitude and opposite sign.

**Figure 6:**
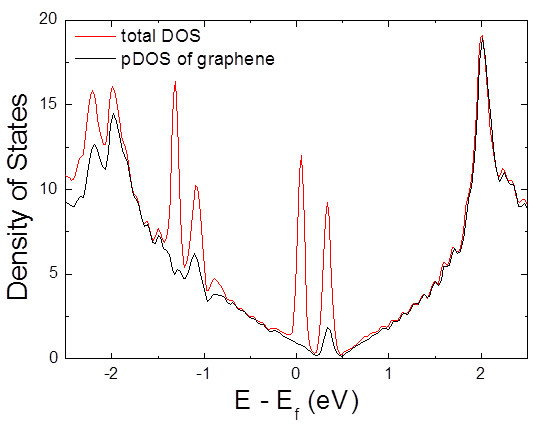
**Total and projected DOS (pDOS) for TCNQ/graphene system.** Red and black lines correspond to total DOS and graphene pDOS, respectively. Fermi level is set to zero.

The extent of charge transfer between TCNQ and graphene was estimated using the Mülliken population analysis based on first-principles calculation. The amounts of charge transfer and adsorption energy [35] for the possible configurations of TCNQ/graphene were summarized in Table 1. Our calculation also supported the limited charge transfer due to strong intermolecular repulsive interaction [35,36]. The effective charge transfer was found to be around 0.47 e per single TCNQ molecule when graphene sheet was sandwiched by two TCNQ molecules with the lowest adsorption energy, although maximum charge transfer amount was only 0.29 e in the case of adsorption on one side. The lowest adsorption energy indicates that adhesion of graphene flakes is improved via interflake TCNQ molecules. These calculation results supported the model of RGO + TCNQ complex films as shown in Figure 3b. The analysis on distribution of the lowest unoccupied molecular level (LUMO) and the highest occupied molecular level (HOMO) suggests that LUMO is delocalized over π orbitals of graphene and HOMO shows strong localization on TCNQ molecule as shown in Figure 5. This confirms that charge transfer between TCNQ and graphene occurs. Furthermore, the electronic states of TCNQ/graphene systems were calculated using the optimized configurations. Total density of states (DOS) of TCNQ/graphene showed clearly strong acceptor levels at 0.3 eV below the Dirac point, resulting in the finite DOS close to the Fermi level. This suggested adsorbed TCNQ depleted the electrons from valence bands of graphene. Another important feature was the projected density of states (pDOS) of graphene around the Dirac point. The pDOS was not significantly affected by the adsorption of TCNQ even though the conductivity of graphene can be reduced by added charged impurities from adsorbed TCNQ as shown in Figure 6. This result does not conflict to the data of electrochemical top-gated transistor study [39].

**Table 1:** Summary of calculation results for TCNQ/graphene charge transfer systems

	**4 × 4**	**6 × 6**	**8 × 8**	**4 × 4 both**	**6 × 6 both**	**8 × 8 both**
Change transfer (e/molecule)	0.16	0.25	0.29	0.26	*0.47*	0.56
Sheet carrier conc. (10^13^ cm^-2^)	1.86	1.32	0.86	3.08	*2.48*	1.68
Distance [Å}	3.06	2.90	3.02	3.11	*2.99*	3.10
Absorption energy (kcal mol^-1^)	-32.91	-38.86	-34.25	-67.72	*-74.86*	-66.14

Values in italics under the 6 × 6 both configuration show the lowest adsorption energy.

**Figure 7:**
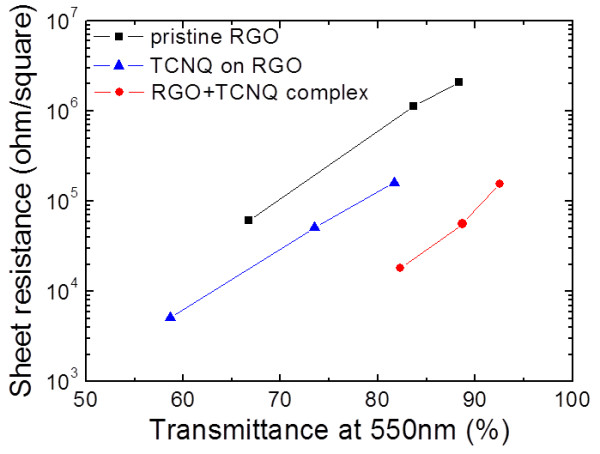
**Sheet resistance of different films as a function of optical transmittance at 550 nm.** Pristine RGO films (black squares), doped RGO films by surface adsorption (blue triangles), and RGO + TCNQ complex films (red circles). The sheet resistance of the RGO + TCNQ complex films decreased drastically by two orders of magnitude, without degradation of optical transparency, which was a more drastic change than the case of doping by surface adsorption.

We verified the effects of doping on the electrical properties of graphene, and demonstrated the huge potential of transparent conductive graphene films. No dominant changes were observed in the optical transmittance spectra after doping, except for the appearance of a slight adsorption around 500 nm by TCNQ molecules [27]. The sheet resistance, *R*_*s*_, as a function of transmittance at 550 nm is summarized in Figure 7. Due to carrier doping via the CT interaction from TCNQ, the sheet resistance of the RGO + TCNQ complex films drastically decreased by two orders of magnitude without significant degradation of the optical transparency as a result of increasing the sheet carrier density from 1.02 × 10^10^ cm^-2^ to 1.17 × 10^12^ cm^-2^ estimated from Hall measurement. Doping stability with time evolution at room temperature under ambient atmosphere was monitored. *R*_*s*_ increased by less than 10% after 1 year, whereas it increased by up to 40 % after 20 days in the case of AuCl_3_ which showed one of the highest doping effect [19]. Thermal stability of our doped films was examined by stepwise annealing from 100°C to 250°C under vacuum. The doping effect was preserved after annealing even at 250°C without any remarkable degradation. This result indicates higher thermal stability than F4-TCNQ [34]. Those stabilities are quite critical issue of doping technique in any application fields. Finally, our chemical doping method was tried by dipping chemical vapor deposition (CVD) graphene purchased from Graphene Platform, Inc. (Houston, TX, USA) in radicalized TCNQ in order to show that our method can be adapted also for CVD graphene. The sheet resistance of the doped CVD graphene decreased to 400 Ω from 1.2 kΩ at 97% of optical transparency. Our doping method exhibits the compatibility with the CVD graphene-based transparent conductive films.

## Conclusions

We developed a novel method for the carrier doping of graphene using radical-assisted conjugated organic molecules in the liquid phase. The absorbance data and the Raman spectra results indicated strong charge transfer interactions between RGO and TCNQ. The high doping efficiency of our method was demonstrated as an improvement in sheet resistance by two orders of magnitude, without degradation of the optical transparency. First-principles calculation predicted the model of our doping mechanism and the origin of high doping efficiency. Furthermore, the doping effect was quite chemically stable. The doped chemically derived graphene films fabricated by all-wet process have huge potential as an alternative material for transparent conductive films in low-cost and low-temperature processes.

## Competing interests

The authors declare that they have no competing interests.

## Authors’ contributions

RI designed and conducted all experiments and characterization and drafted the manuscript. PK, MB, and YK helped in technical support for experiments and drafting the manuscript. Both AS and MK have read and approved the final manuscript. All authors read and approved the final manuscript.
